# Evaluation of the performance of Ca-deficient hydroxyapatite (CDHA)/MgF_2_ bilayer coating on biodegradable high-purity magnesium in a femoral condyle defect model in rabbits

**DOI:** 10.1093/rb/rbac066

**Published:** 2022-10-04

**Authors:** Shibo Huang, Junlei Li, Kairong Qin, Zhiqiang Wang, Jiahui Yang, Fang Cao, Weirong Li, Yupeng Liu, Lipeng Liu, Dewei Zhao

**Affiliations:** Faculty of Electronic Information and Electrical Engineering, School of Biomedical Engineering, Dalian University of Technology, No. 2 Linggong Road, Ganjingzi District, Dalian, Liaoning 116024, China; Orthopaedic Department Affiliated Zhongshan Hospital of Dalian University, No. 6 Jiefang Street, Zhongshan District, Dalian, Liaoning 116001, China; Orthopaedic Department Affiliated Zhongshan Hospital of Dalian University, No. 6 Jiefang Street, Zhongshan District, Dalian, Liaoning 116001, China; Department of Optoelectronic Engineering and Instrumentation Science, Dalian University of Technology, No. 2 Linggong Road, Ganjingzi District, Dalian, Liaoning 116024, China; Orthopaedic Department Affiliated Zhongshan Hospital of Dalian University, No. 6 Jiefang Street, Zhongshan District, Dalian, Liaoning 116001, China; Orthopaedic Department Affiliated Zhongshan Hospital of Dalian University, No. 6 Jiefang Street, Zhongshan District, Dalian, Liaoning 116001, China; Faculty of Electronic Information and Electrical Engineering, School of Biomedical Engineering, Dalian University of Technology, No. 2 Linggong Road, Ganjingzi District, Dalian, Liaoning 116024, China; Orthopaedic Department Affiliated Zhongshan Hospital of Dalian University, No. 6 Jiefang Street, Zhongshan District, Dalian, Liaoning 116001, China; Dongguan Eontec Co., Ltd, Songshan Lake Science and Technology Industrial Park, Dongguan, Guangdong 523000, China; Orthopaedic Department Affiliated Zhongshan Hospital of Dalian University, No. 6 Jiefang Street, Zhongshan District, Dalian, Liaoning 116001, China; Orthopaedic Department Affiliated Zhongshan Hospital of Dalian University, No. 6 Jiefang Street, Zhongshan District, Dalian, Liaoning 116001, China; Orthopaedic Department Affiliated Zhongshan Hospital of Dalian University, No. 6 Jiefang Street, Zhongshan District, Dalian, Liaoning 116001, China

**Keywords:** high-purity magnesium, MgF_2_, Ca-deficient hydroxyapatite, femoral condyle

## Abstract

The two most critical factors in promoting the clinical translation of magnesium (Mg) are reducing its degradation rate and improving its osteogenesis. In this study, a Ca-deficient hydroxyapatite (CDHA)/MgF_2_ bilayer coating was prepared on high-purity magnesium (HP Mg) rods by fluorination and hydrothermal treatment. Scanning electron microscope showed that the thickness of the bilayer coating was 3.78 μm and that the surface morphology was nanoscale. In an *in vivo* experiment on femoral condyle defects in rabbits, the serum magnesium ion levels of rabbits were always in the normal range after surgery, and the liver and kidney functions were not abnormal, which indicated that the CDHA/MgF_2_ bilayer coating has good biosafety. Micro-CT showed that the CDHA/MgF_2_ bilayer coating significantly reduced the degradation rate of the HP Mg rods and enhanced the promotion of bone formation. Hard tissue sections showed that the CDHA/MgF_2_ bilayer coating gave the bone tissue a tight contact interface with the HP Mg rod and improved the bone mass. Immunohistochemistry showed that the expression of vascular endothelial growth factor and BMP-2 was more obvious. These results confirm that the CDHA/MgF_2_ bilayer coating can improve the properties of HP Mg and provide a basis for the further transformation of HP Mg in the future. It also provides a new reference for the surface modification of magnesium metal.

## Graphical Abstract

**Figure:**
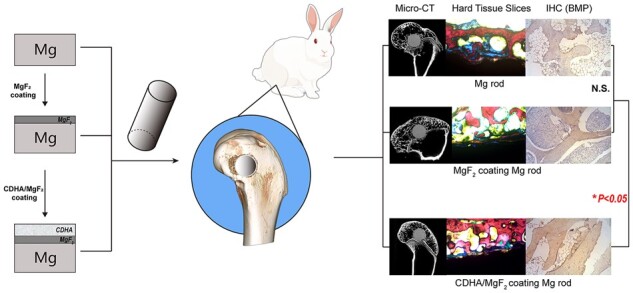

## Introduction

The good biocompatibility of magnesium metal has been widely confirmed [1, 2]. Therefore, the transformation of magnesium has always been a topic of great interest. In bone tissue engineering, degradable magnesium metal can not only degrade *in situ* but also promote the formation of new bone [3, 4]. Moreover, the elastic modulus of magnesium is similar to that of human cortical bone, preventing stress shielding. Therefore, magnesium metal has the potential to become a new generation of orthopaedic implant materials [5, 6]. Although magnesium metal has many advantages, an interfacial gap exists between the metal and the surrounding bone tissue after degradation because the rate of new bone formation does not match the degradation rate of magnesium [7–9]. To meet the needs of an increasing number of orthopaedic applications, it is important to further improve the properties of magnesium metal. Therefore, reducing the initial degradation rate of magnesium metal and increasing the induction of new bone are the keys to improving its performance. Compared with magnesium alloy, the composition of high-purity magnesium (HP Mg) is simple. To avoid changing the composition of the material, coating the surface of HP Mg is the best choice.

At present, commonly used surface modification technology mainly includes microarc oxidation and deposition (including electrodeposition and bionic deposition) [10–12]. A satisfactory magnesium coating should have ideal biocompatibility, a reduced rate of degradation and the ability to promote bone formation and vascularization [13–15]. A large number of reports show that a fluorinated layer bonds firmly to magnesium metal and significantly decreases the degradation rate [16, 17]. However, it is not clear whether fluorinated coatings have osteogenic effects. Jiang *et al.* [18] reported that MgF_2_ showed better new bone formation than bare Mg. However, Barbeck *et al.* [19] reported that the difference in new bone formation between the MgF_2_ and bare Mg groups was not statistically significant. In addition, the MgF_2_ coating is thin and has a limited protective effect on the matrix. At present, fluorine treatment is usually used as a pre-treatment process to prepare composite coatings. Calcium–phosphorus coating is the most commonly used coating technology at present and includes hydroxyapatite, dicalcium phosphate, etc. It can promote the formation of new bone and reduce the degradation rate of magnesium [20]. However, the bonding force between the calcium–phosphorus coating and the magnesium metal surface is weak, and the coating easily falls off. Our team synthesized the advantages of the fluorinated coating and calcium–phosphorus coating and prepared a CDHA/MgF_2_ bilayer coating on the Mg surface by combining fluorination treatment with hydrothermal treatment [21]. *In vitro* experiments showed that the CDHA/MgF_2_ coating exhibits nanoscale roughness under electron microscope and adheres well to magnesium metal. The CDHA/MgF_2_ bilayer coating can significantly promote the adhesion, proliferation and differentiation of MG63 cells and exhibits improved corrosion resistance [21].

A large number of clinical studies have confirmed that the safety and performance of biomaterials differ between *in vivo* and *in vitro* studies. Therefore, animal testing is crucial. Limited by the experimental environment, it is difficult to simulate changes in stress and body fluid *in vitro*, so the performance of coatings *in vivo* cannot be accurately evaluated *in vitro* [4]. Compared with *in vitro* experiments, animal experiments can provide simulated conditions that are closer to the human physiological environment. Moreover, the results obtained from animal tests can predict the transmission of information to the human clinical environment. Therefore, it is very important to accurately evaluate the safety, osteogenesis, compatibility and other animal tests of materials before clinical transformation and application.

In this study, we constructed CDHA/MgF_2_ bilayer coatings on HP Mg rods. Scanning electron microscope (SEM) was carried out to characterize the microstructure and composition of the CDHA/MgF_2_ bilayer coating. A rabbit model of femoral lateral condyle defects was constructed. The bone defects were filled with HP Mg rods, MgF_2_-coated rods and CDHA/MgF_2_ bilayer-coated rods. By comparing the performance of the three types of magnesium rods in bone defects, the safety, degradation and osteogenesis of the bilayer coating *in vivo* were evaluated.

## Materials and methods

HP Mg rods (≥99.99 wt%) were obtained from Dongguan Eontec Co., Ltd. The initial HP Mg rods were processed to 5 mm in diameter and 10 mm in length. All magnesium rods were ground with 400, 800, 1200 and 2000 grit SiC sandpaper in turn. After ultrasonic cleaning with ethanol and distilled water for 5 min, the samples were dried with hot air.

First, the surface of the HP Mg rod was fluorinated. HP Mg bars were treated with hydrofluoric acid (48 wt%) at 25°C for 24 h. After 24 h, they were rinsed thoroughly with deionized water and air dried. An HA coating was then prepared on the surface of the magnesium fluoride coating by hydrothermal treatment in a 50 ml Teflon inner autoclave [22]. A 35 ml aqueous solution containing 0.25 M ethylenediamine tetra-acetate calcium disodium hydrate (Ca-EDta, Sigma Aldrich, USA) and potassium hydrogen phosphate (KH_2_PO_4_, Sigma Aldrich, USA) was added to an autoclave, and the pH of the solution was adjusted to 8.9 with 5 M sodium hydroxide solution. The Mg rods coated with magnesium fluoride were immersed in the solution for 6 h to form HA/MgF_2_ double-coated structures on the HP Mg rods.

### Coating surface analysis

The surface of the bilayer rods was observed by a scanning electron microscope (SEM, Hitachi 3500). The elemental composition of the coatings was determined by energy dispersive spectroscopy (EDS, 7401 EDX).

### Surgical procedure

**Figure 1.**
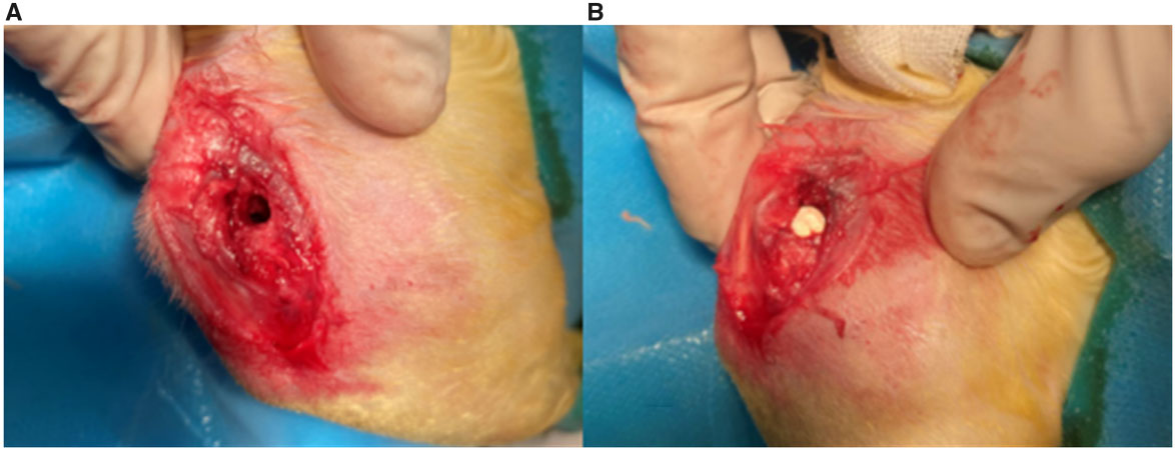
Operation of femoral condyle defect in rabbit: (**A**) Construction of femoral condylar defect; (**B**) Femoral condylar defect was filled with magnesium rod.

A total of 45 rabbits (weight 2–2.5 kg, 3 months old, no sex limitation) were included in this study. Forty-five rabbits were randomly divided into three groups: HP Mg groups, MgF_2_ coating groups and CDHA/MgF2 bilayer groups. All rabbits underwent femoral condyle defect surgery (Fig. 1). Bone defect models were constructed in 45 rabbits with unilateral femoral condyles. At 4, 8 and 12 weeks after surgery, 15 rabbits (5 in each group) were randomly selected from the three groups and sacrificed. The rabbit was anaesthetized by Lu Mian Ning and placed in the lateral position. Sterile surgical towels were laid out after routine disinfection. The skin and subcutaneous fascia were sequentially incised on the lateral condyle of the femur. Blunt separation of the gracilis muscle and vastus medialis muscle was performed through the muscular space. The lateral femoral condyle was exposed. The bone defect was first drilled with a 2.5 mm diameter drill bit, and then the bit size was gradually increased to 5 mm to make a bone defect model of the lateral wall of the femoral lateral condyle with a diameter of 5 mm and a depth of 10 mm. Magnesium rods were implanted into the bone defect and sutured subcutaneously, and then the skin was sutured. Rabbits were awakened by intramuscular injection by Lu Xing Ning. Infection was prevented by gentamicin injection for 3 days.

### *In vivo* safety

Whole blood (2–3 ml) was extracted through the rabbit ear margin vein for analysis. The extracted blood was centrifuged at 1200 RPM/min for 7 min, and the upper serum was separated. The serum magnesium ion concentration was measured by ICP–MS. Alanine aminotransferase (ALT), alanine aminotransferase (AST), blood urea nitrogen (BUN) and creatinine (CR) were measured by an automatic biochemical-immune analyser (Roche Cobas 6000, USA).

### Micro-CT image analysis

The rabbits undergoing surgery were sacrificed to obtain micro-CT scans at 4, 8 and 12 weeks after surgery. All condyles of femur samples were scanned with Inveon Micro-CT produced by Siemens (Berlin, Germany) at medium to high resolution. Image scanning was performed at 80 kV and 500 μA standard. The effective pixel size was 15.48 μm. Three-dimensional reconstruction of the femoral condyle was performed with software. The volume of interest at the top of the pure magnesium rod was selected from these regions for three-dimensional reconstruction. The bone volume/total volume (BV/TV) and trabecular bone number (Tb.N) were determined and analysed. Since the thickness of the bilayer coating is only 3–4 μm, the influence on the volume of the HP Mg rod can be ignored. Micro-CT was used to perform 3D reconstruction of the magnesium rod, and then the volume was measured and the changes in appearance were observed. The degradation rate of the Mg rod was evaluated by comparison with the initial magnesium rod volume. Degradation rate (%) = 100% × (mean volume of original HP Mg rods-mean volume of HP Mg rods at each time point)/mean volume of original HP Mg rods.

### Histology and immunohistochemistry

After micro-CT scanning, the samples were immobilized in formalin for 5 days, dehydrated in a gradient of 75–100% ethanol solution, and embedded in methyl methacrylate. The treated femoral condyle samples were sliced with a hard tissue slicer diamond saw (EXAKT, Germany) (approximately 200 μm thick), ground to 50 μm thick with a grinding machine, and stained with picric acid magenta (van Gieson staining). Hard tissue sections were observed using a standard light microscope (Leica LA Microsystems, Bensheim, Germany), and osteogenesis was qualitatively analysed. Positive areas were quantified with the Image Pro Plus software (Version 6.0, Media Cybernetics, USA) based on the histology images. All tests were collected with five specimens.

Rabbit femoral condyle samples were embedded in paraffin for tissue sectioning (Leica Slicer). To analyse the angiogenic and osteogenic potential of the femoral condyle, bone morphogenetic protein-2 (BMP-2) (rabbit antibody, diluted 1:2000, Abcam, Cambridge, MA, UK) and vascular endothelial growth factor (VEGF)-specific antibodies (rabbit antibody, diluted 1:1000, Abcam, Cambridge, MA, UK) were used for immunohistochemical analyses. To block endogenous peroxidase activity, slices were immersed in 0.01 M phosphate-buffered saline (PBS) with 3% hydrogen peroxide for 10 min and then washed with PBS. The sections were placed in 10% rabbit normal antiserum (Solarbio, Beijing, China) for 30 min, treated with primary antibody at 4°C overnight, and incubated with enzyme-labelled secondary antibody for 30 min. To show the immune response, slices were reacted with a solution of diaminobenzidine in the dark. Finally, the slices were hematoxylin-stained and mounted. Negative controls were slices without primary antibody processing. Each test was performed three times. The portions were photographed and observed. To objectively examine and determine the mean integral optical density (IOD) colour and optical density correction for all photos, Image Pro Plus 6.0 software was utilized. The total positive area and positive total integrated optical density of all images were quantified using the same positive expression analysis standard.

Mean IOD = Positive total integrated optical density (IOD)/total positive area (Area).

### Statistical analysis

All results are expressed as the means ± standard deviations (SD). Two-way analysis of variance (ANOVA) was used for statistical analysis. *P* < 0.05 indicated that the difference was statistically significant.

## Results and discussion

### CDHA/MgF2 bilayer coating morphology characteristics

**Figure 2.**
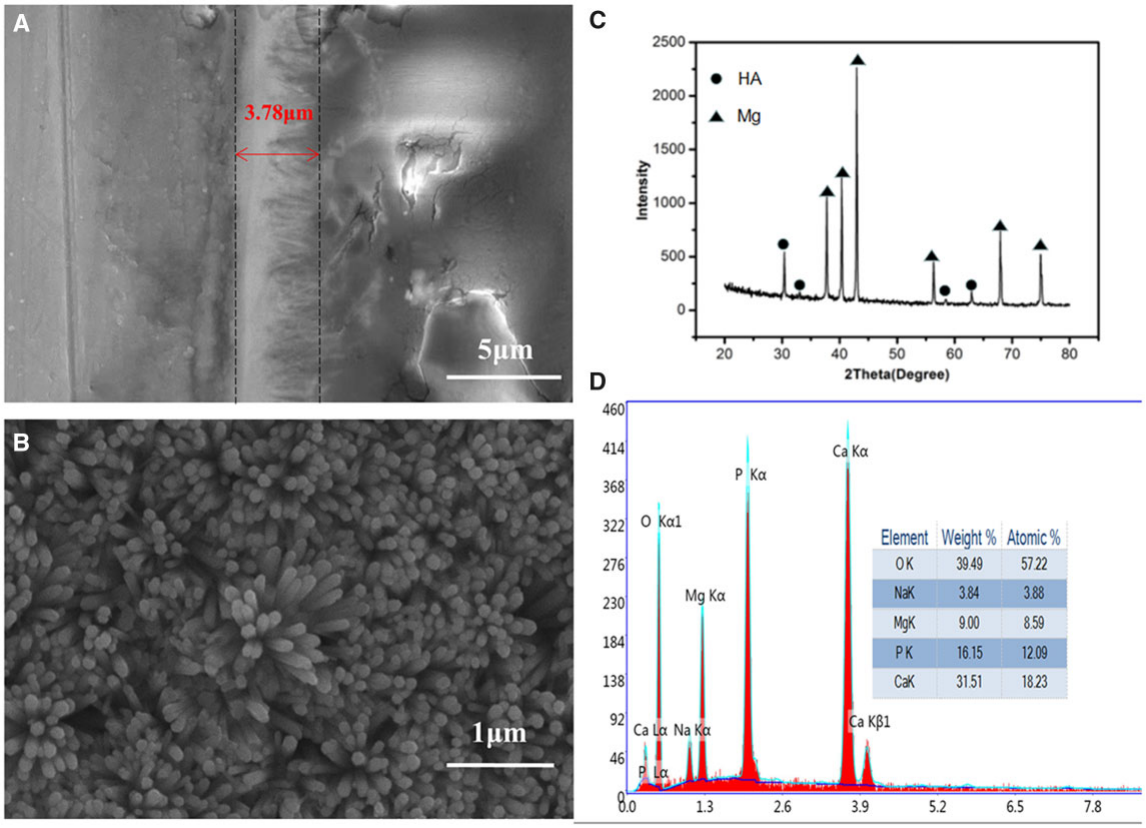
(**A**) The thickness of the CDHA coating (the black dashed area shows the CDHA coating); (**B**) Nanorod-like HA crystals on the surface of CDHA/MgF_2_ were observed; (**C**) XRD patterns of CDHA/MgF_2_ coating; (**D**) Element components of the CDHA/MgF_2_ bi-layer.

The thickness of the CDHA coating is approximately 3.78 μm (Fig. 2A). According to our previous study, the fluoride layer thickness is about 2 μm [21], and the CDHA/MgF_2_ coatings thickness is about 5–6 μm. The surface of CDHA/MgF_2_ is composed of nanorod-like HA crystals (Fig. 2B). Typical Mg peaks can be observed in the XRD patterns of CDHA/MgF_2_ (Fig. 2C). EDS analysis showed that the coating contains Mg, Na, Ca, O and P (Fig. 2D). Mg^2+^ and Na^+^ ions may take the place of Ca^2+^. Through calculation, the ratio of calcium and phosphorus is approximately 1.51. This result was lower than the traditional stoichiometry of hydroxyapatite (1.67). This indicates that we formed a calcium-deficient HA (CDHA, Ca/P atomic ratio between 1.33 and 1.55) coating on the Mg substrate. Our previous basic research results [21] show that when a CDHA coating is directly combined with a magnesium metal matrix, the binding force between the coating and the matrix is low (10.9 MPa), whereas when MgF_2_ is used as an intermediate layer, the binding force of the CDHA coating can be significantly improved (14.9). A higher binding force can reduce the peeling of the coating on the magnesium metal surface caused by friction during implantation to prevent local rapid corrosion of the exposed magnesium metal matrix. This is very important for improving the surrounding microenvironment of the implant at the initial stage of implantation because a highly alkaline environment will activate osteolysis and inhibit osteogenesis, thus affecting the regeneration of surrounding bone tissue.

### *In vivo* safety

Animal experiments play an important role in clinical translational research. Experimental results can be used to provide an information reference for human clinical trials, and the results will determine whether clinical trials can be continued. The safety of biomaterials *in vivo* is always a priority. Magnesium is the fourth most abundant metal ion in the human body after potassium, sodium and calcium, and its content is 0.3–0.4 g/kg. *In vivo*, 66% of magnesium is found in bones and teeth, 33% in cells, and only approximately 1% in extracellular body fluids and blood. Serum contains less than 0.3% of the total magnesium. Magnesium plays an important role in various biological metabolic processes, participating in energy metabolism, glycolysis and nucleic acid and protein synthesis, especially as a cofactor of adenosine triphosphate. It also regulates the flux of potassium, calcium and zinc through cell membranes. It is involved in various aspects of enzyme metabolism; in the health of the brain, cardiac muscle, skeletal muscle and bone; and in many other physiological activities. In this study, the serum magnesium ion concentration, liver function and renal function of the HP Mg group, MgF_2_ group and CDHA/MgF_2_ group were measured preoperatively and 1 day, 3 days, 4 weeks, 8 weeks and 12 weeks after surgery. Alanine aminotransferase (ALT) and aspartate aminotransferase (AST) were used to reflect liver function. BUN and CR were selected to reflect renal function. Due to concerns about magnesium accumulation caused by the rapid degradation of materials at the initial stage of implantation, 1 and 3 days time points were added for measurement. The serum magnesium ion concentrations in the three groups were all normal. The serum Mg ion concentration in the CDHA/MgF_2_ group was not significantly higher than that in the other groups. The indexes of liver function and kidney function were in the normal ranges and showed no abnormal changes in the three groups. These results indicate that the CDHA/MgF_2_ bilayer coating exhibits good biosafety *in vivo*.

### 3D Reconstruction of the femoral condyle

**Figure 3.**
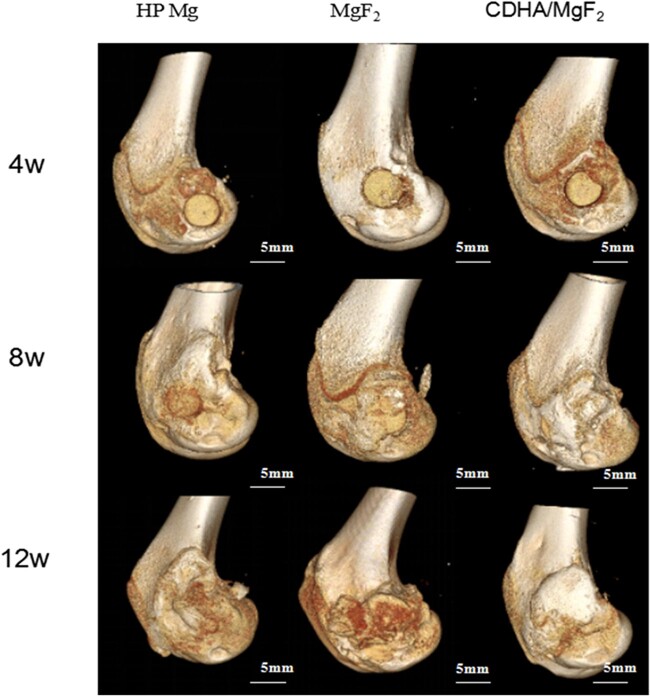
The femoral condyles were reconstructed at 4, 8 and 12 weeks after surgery. With the prolongation of time, the defect surface of femoral condyle was gradually covered by new bone, and the CDHA/MgF_2_ group showed better new bone formation ability.

Animal models are mainly divided into large animals and small animals. Small animal models are generally used to evaluate the preliminary properties of materials. Large animal models are needed for comprehensive evaluation before clinical trials. In this study, a rabbit model of femoral condyle defects was selected to preliminarily study the performance of the CDHA/MgF_2_ double coating. Mice and rabbits are the most common small animal models and have the advantages of low unit cost, ease of feeding, many kinds of experimental antibodies and ease of purchase. Small bone defects can heal themselves, so large bone defect models should be constructed to ensure the accuracy of the experiment. The femoral condyle area of rabbits is larger than that of rats, so a large bone defect model can be constructed to evaluate the material. In this study, a bone defect with a diameter of 5 mm was constructed to eliminate the possibility of bone defect self-healing. At 4 weeks after surgery, filled magnesium rods could be seen in the bone defects of the femoral condyles in all three groups. Some new bone tissue was generated on the surface of the bone defects in the CDHA/MgF_2_ bilayer coating rod group. The HP Mg group and MgF_2_ group had no obvious bone formation on the defect surface. At the initial stage of magnesium rod implantation, the local high alkaline environment is not conducive to new bone formation. The osteogenic activity of MgF_2_ is weak, so early group samples revealed no obvious new bone formation around the newly implanted magnesium and MgF_2_ rods. The CDHA coating, in contrast, can protect the magnesium substrate, preventing the formation of a highly alkaline environment around new implants. At the same time, the CDHA coating itself has good biological activity in bone conduction and bone induction, which contributes to the formation of new bone around the magnesium rod after implantation. The surface of the femoral condyle defect in the CDHA/MgF_2_ bilayer group was completely covered with new bone at 8 weeks after surgery. Similar bone growth was observed in the HP Mg group and in the MgF_2_ rod group. The surface of the femoral condyle defect was covered with new bone in the three groups at 12 weeks after surgery. With the extension of implantation time, the surface of the HP Mg and MgF_2_ rod samples was covered by magnesium oxide, hydroxide, phosphate deposits of calcium from the human body and adsorbed proteins. The degradation rate of the samples decreased, and new bone could be formed on the surface of the implants. These results indicate that the CDHA/MgF_2_ bilayer coating group showed better bone defect better than the HP Mg and the MgF_2_ group (Fig. 3).

### The 3D reconstruction and degradation of Mg rods

**Figure 4.**
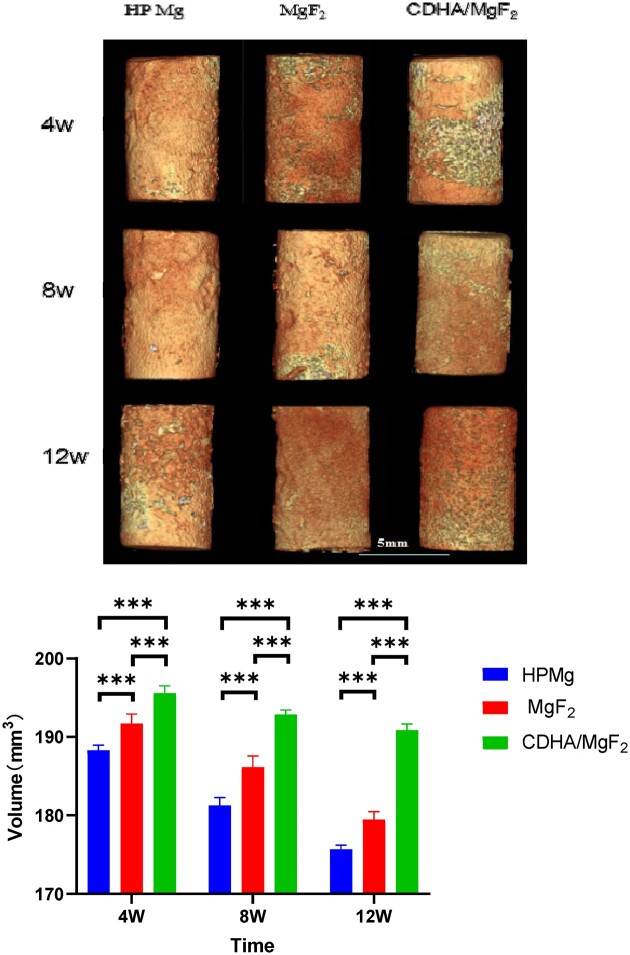
Mg Rods were reconstructed in the HP Mg, MgF_2_ and CDHA/MgF_2_ groups at 4, 8 and 12 weeks after surgery. Different degrees of degradation occurred on the surface of the magnesium rods in the three groups. The volume change of rods of HP Mg, MgF2 and CDHA/MgF_2_ groups at 4, 8 and 12 weeks after surgery. Data are presented as the mean ± SD. *P < 0.05,**P < 0.01,***P < 0.001.

No obvious change was observed on the surface of the CDHA/MgF_2_ bilayer-coated rod at 4 weeks after surgery. Slight corrosion was not observed on the surface of the CDHA/MgF_2_ bilayer coating rod until 12 weeks after surgery. In the HP Mg group, degradation of the rod surface was observed at 4 weeks after surgery. At 12 weeks after surgery, obvious corrosion of the rod was observed. In the MgF_2_ group, the corrosion of the rod surface became obvious by 8 weeks after surgery. The CDHA/MgF_2_ bilayer coating rod degraded by approximately 0.4%, 1.8% and 2.8% at 4, 8 and 12 weeks after surgery, respectively. The MgF_2_ coating rod degraded by approximately 2.3%, 5.2% and 8.6% at 4, 8 and 12 weeks after surgery, respectively. The HP Mg rod degraded by approximately 4.1%, 7.7% and 10.5% at 4, 8 and 12 weeks after surgery, respectively (Fig. 4). Because the biochemical environment *in vivo* is highly complex, it is very important to simulate this complexity *in vitro* and obtain accurate data on degradation behaviour *in vivo*. Therefore, it is very important to evaluate the degradation of biomaterials *in vivo*. The initial mass of the magnesium rod used for *in vivo* animal experiments was approximately 0.34 g. According to the change in magnesium metal volume measured by micro-CT, the amounts of magnesium ion released by the degradation of magnesium metal in the HP Mg group, MgF_2_ group and CDHA/MgF_2_ bilayer coating group were approximately 0.348 mg, 0.404 mg and 0.113 mg per day, respectively. This value was far lower than the recommended daily intake of magnesium ion (3.6 mg/d kg), which further verified the biosafety of the HP Mg group, MgF_2_ group and CDHA/MgF_2_ bilayer coating group for degradation in animals. In clinical translation, the degradation of magnesium metal is also related to the body fluids and stress at the implantation site. Increased stress and rich exchange of body fluids will accelerate the corrosion of magnesium. Therefore, it is very important to ensure a low degradation rate of magnesium in the initial stage of implantation. Previous literature has reported that fluorination can significantly reduce the degradation rate of magnesium [23]. Wang *et al.* [24] reported that magnesium substrate degradation in the MgF_2_ group was faster than that in the Ca-coated group, and more H_2_ was produced along with the degradation. In this study, the degradation rate of the MgF_2_ rod was observed to be significantly lower than that of the HP Mg rod. Compared with that of the MgF_2_ group, the degradation rate of the CDHA/MgF_2_ bilayer coating was further reduced, indicating that the CDHA/MgF_2_ bilayer coating provides better protection and can better reduce the degradation rate of HP Mg rods. We believe that this is because even if the surface coating disappears prematurely, there is still a fluorinated layer beneath that can effectively protect the HP Mg rod. The decreases in Mg volume were directly linked to the generation of natural hydrogen gas. The degradable magnesium alloy can be removed by local blood flow when it degrades and produces a small amount of hydrogen during implantation. Therefore, the reduction in degradation speed will also reduce the rate of hydrogen generation to allow full absorption with surrounding tissue exchange and prevent adverse effects.

### New bone formation *in vivo*

**Figure 5.**
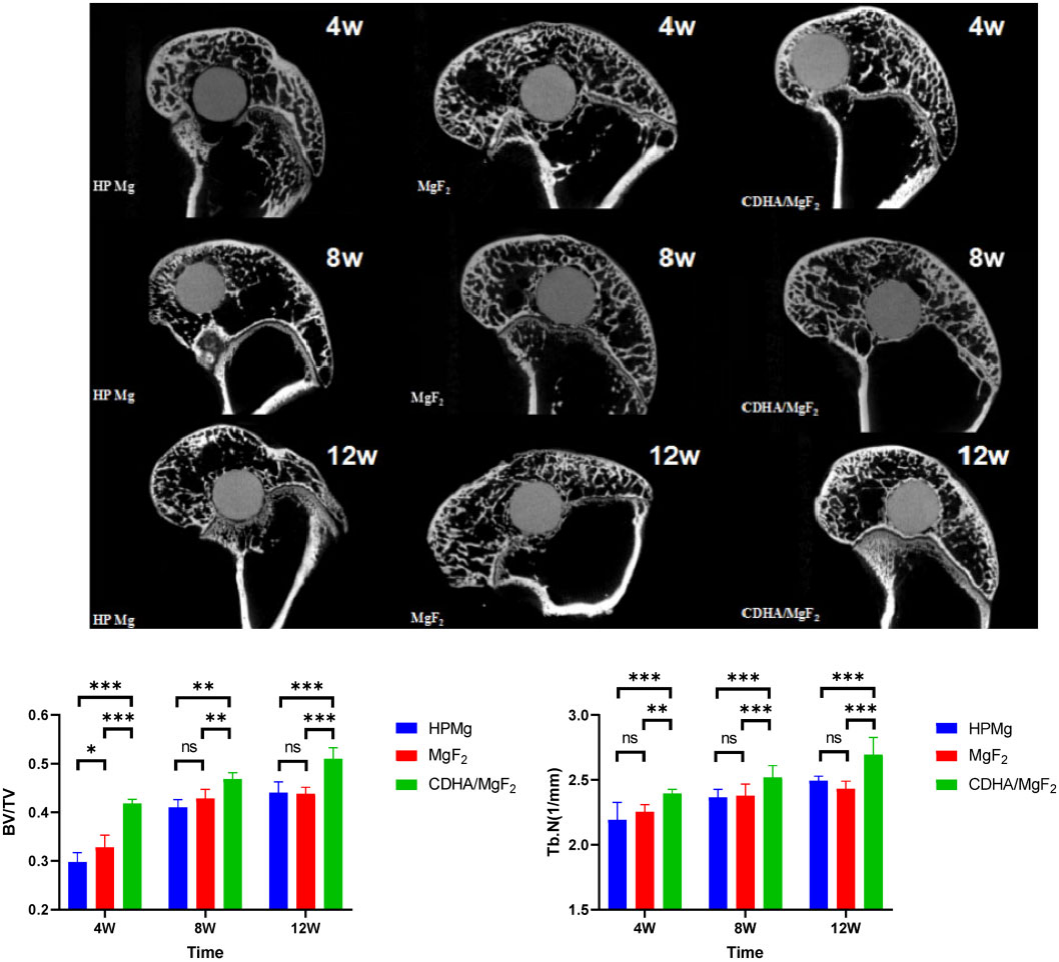
The micro-CT scan of HP Mg, MgF_2_ and CDHA/MgF_2_ groups at 4, 8 and 12 weeks after surgery. The change of the BV/TV and Tb.N among three groups. Data are presented as the mean ± SD. ns: *P* > 0.05, *P < 0.05, **P < 0.01, ***P < 0.001.

Micro-CT scans showed new bone formation on the surface of the Mg rods in all three groups at 4, 8 and 12 weeks after surgery. Moreover, there was no bone dissolution during the degradation of Mg rods in the three groups. Compared with the other two groups, the Mg rods in the CDHA/MgF_2_ bilayer coating group adhered more closely to the surrounding bone tissue. Especially at 12 weeks after surgery, significant bone tissue was observed surrounding the magnesium rod (Fig. 5). At 4 weeks after surgery, the MgF_2_ group had higher BV/TV and Tb.N than the HP Mg group, but there was no difference at 8 and 12 weeks after surgery. At 4, 8 and 12 weeks after surgery, the CDHA/MgF_2_ bilayer group had considerably higher BV/TV and Tb.N than the other two groups. Wang *et al.* found that the BV/TV in the Ca-P-coated Mg group was considerably higher than that in the MgF_2_ group at 4 and 8 weeks after surgery. The results showed that the calcium-phosphorus coating promoted bone formation more effectively than the fluorinated. Similar to our research results, Wang *et al.* [24] implanted calcium and phosphorus-coated scaffolds and magnesium fluoride scaffolds into the rat femoral defect model at the same time, and the experimental results showed that the BMD and BV/TV of magnesium alloy coated with calcium and phosphorus were significantly superior to those obtained by using Mg–Nd–Zn–Zr magnesium alloy with a fluorinated layer by micro-CT scanning. This indicates that the calcium–phosphorus coating has a stronger osteogenic effect than the fluorinated coating. In this study, the BV/TV of the MgF_2_ group was better than that of the HP Mg group at 4 weeks after surgery, but there was no difference between the two groups at 8 and 12 weeks after surgery. This indicates that the osteogenic effect of the fluoride coating is no longer obvious after 8 weeks. The CDHA/MgF_2_ group maintained the promotion of new bone formation for 12 weeks after surgery. BV/TV and Tb.N were significantly better than those in the other two groups, indicating that the CDHA/MgF_2_ bilayer group promoted new bone formation more effectively.

### Histological and immunohistochemical analysis

**Figure 6.**
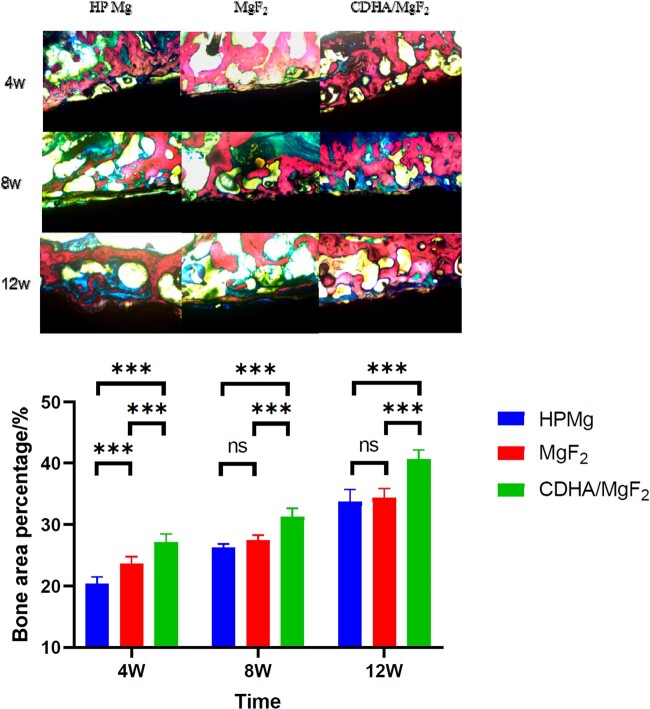
The hard tissue section of HP Mg, MgF_2_ and CDHA/MgF_2_ groups at 4, 8 and 12 weeks after surgery (40× magnification). Hard tissue sections were stained with picric acid magenta (van Gieson staining). Data are presented as the mean ± SD. ns: *P* > 0.05, *P < 0.05, **P < 0.01, ***P < 0.001.

At 4 weeks after surgery, the hard tissue sections showed that the Mg rod in the HP Mg group began to degrade, and there was new bone tissue on the surface. There was a space between some of the bone tissue and the magnesium rod surface. This interfacial gap was still present at 8 weeks and nearly disappeared at 12 weeks. At 4 weeks after surgery, the MgF_2_ group had new bone tissue and magnesium rod adhesion performance outperforming those of the HP Mg group, and the amount of bone tissue at 8 and 12 weeks after surgery was similar to that of the HP Mg group. In the CDHA/MgF_2_ bilayer group, the new bone tissue was tightly applied with a magnesium rod at 4 weeks after surgery, and the new BV was the largest. At 8 and 12 weeks after surgery, the bone tissue was still tightly attached to the magnesium rod, and the bone mass increased with time. Compared with the other two groups, the CDHA/MgF_2_ bilayer group consistently showed the best interface and new bone formation after surgery. The literature reported [25, 26] that by increasing osteoblast proliferation, Ca-P coatings could aid in the creation and maturation of bone at the implant interface. Our previous study also confirmed that a CDHA/MgF_2_ bilayer coating can promote the adhesion of osteoblasts [21]. This result was consistent with the osteogenic effect of the CDHA/MgF_2_ bilayer coating in this *in vivo* study (Fig. 6).

**Figure 7.**
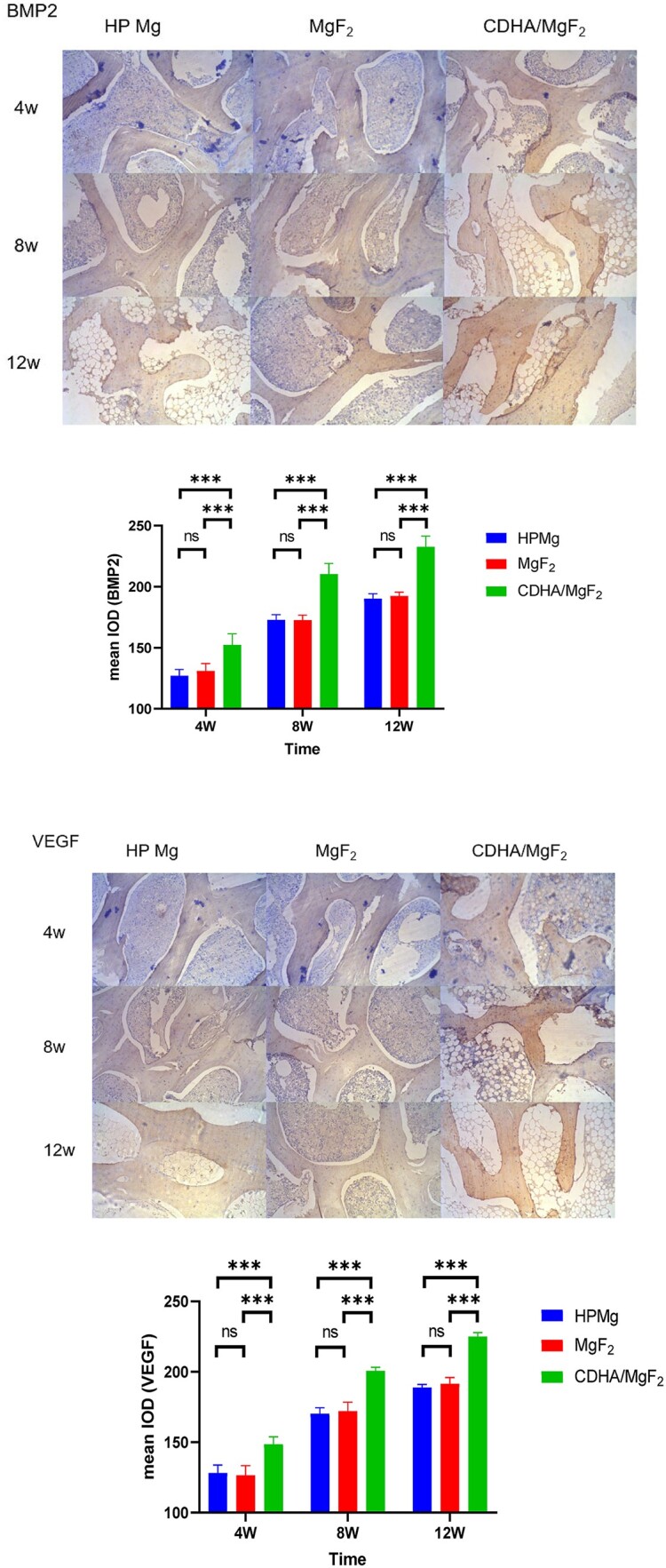
The VEGF and BMP-2 express of HP Mg, MgF_2_ and CDHA/MgF_2_ groups at 4, 8 and 12 weeks after surgery. The slices were hematoxylin-stained and mounted. The positive expression staining shows yellowish brown. Quantitative analysis of BMP-2 and VEGF protein was performed in each time and the mean IOD was calculated. Data are presented as the mean ± SD. *P < 0.05, **P < 0.01, ***P < 0.001.

Here, we used two antibody chromogenic agents, BMP-2 and VEGF, to determine the expression of cell antigens in tissues. BMP-2 and VEGF are secretory proteins that are secreted by cells to promote the expression of new bone and angiogenesis. The positive expression staining is yellowish brown. BMP-2 plays an important role in osteogenic induction, and the osteogenic potential of osteoblasts is reflected in BMP-2 content [27]. VEGF promotes blood regeneration and vascular mitosis, as well as increasing vascular endothelial cells and vascular permeability. Furthermore, by promoting angiogenesis, VEGF has been shown to enhance bone repair and regeneration. Sun *et al.* [28] implanted tiny screws containing tiny fluoride coatings AZ31B alloy screws into the rabbit's mandible and femur. The tiny screws showed better expression of BMP-2 than tiny titanium screws. These results indicate that fluorinated magnesium alloy promotes BMP-2 expression more effectively than titanium alloy. Chen *et al.* [29] showed that calcium- and phosphorus-coated magnesium scaffolds can significantly upregulate VEGF and BMP-2 expression, activate the M2 macrophage phenotype, release BMP-2, and accelerate bone regeneration. Saghiri *et al.* [30] showed that calcium ions and phosphorus ions also participated in promoting angiogenesis. Wang *et al.* [31] found that compared with MgF_2_-coated magnesium alloy scaffolds, calcium phosphate-coated magnesium alloy scaffolds showed superior angiogenic ability. In this study, positive expression of BMP-2 and VEGF was found in the three groups at 4, 8 and 12 weeks after surgery. Similar positive expression was found in the HP Mg group and MgF_2_ group. Compared with the other two groups, the expression of the CDHA/MgF_2_ bilayer group was more obvious in the three groups, and the expression was strongest at 12 weeks after surgery (Fig. 7). This also showed that the CDHA/MgF_2_ bilayer group had the better potential for promoting bone and vascularization.

## Conclusion

Fluorination and hydrothermal treatment were used to create a CDHA/MgF_2_ bilayer coating on HP Mg rods. The surface morphology of the CDHA/MgF_2_ bilayer coating was nanoscale. The coating can obviously reduce the degradation rate of HP Mg and promote bone and vascularization. The CDHA/MgF_2_ bilayer coating can enhance the properties of HP Mg and provide a basis for its further clinical transformation in the future. It also provides a new reference for the surface modification of magnesium metal.

## Funding

This work was supported by Dalian Health Commission, medical key specialty of Dengfeng project [grant number (2021)243] and National Orthopedics and Sports Rehabilitation Clinical Research Center Innovation Fund (2021-NCRC-CXJJ-ZH-28).

*Conflicts of interest statement*. None declared.
